# Association of Indoor Smoke-Free Air Laws with Hospital Admissions for Acute Myocardial Infarction and Stroke in Three States

**DOI:** 10.1155/2012/589018

**Published:** 2012-06-20

**Authors:** Brett R. Loomis, Harlan R. Juster

**Affiliations:** ^1^RTI International, 3040 Cornwallis Road, Research Triangle Park, P.O. Box 12194, NC 27709, USA; ^2^Bureau of Chronic Disease Evaluation and Research, New York State Department of Health, Corning Tower Room 1084, Empire State Plaza, Albany, NY 12237-0679, USA

## Abstract

*Objective*. To examine whether comprehensive smoke-free air laws enacted in Florida, New York, and Oregon are associated with reductions in hospital admissions for acute myocardial infarction (AMI) and stroke. *Methods*. Analyzed trends in county-level, age-adjusted, hospital admission rates for AMI and stroke from 1990 to 2006 (quarterly) for Florida, 1995 to 2006 (monthly) for New York, and 1998 to 2006 (monthly) for Oregon to identify any association between admission rates and passage of comprehensive smoke-free air laws. Interrupted time series analysis was used to adjust for the effects of preexisting moderate local-level laws, seasonal variation in hospital admissions, differences across counties, and a secular time trend. *Results*. More than 3 years after passage of statewide comprehensive smoke-free air laws, rates of hospitalization for AMI were reduced by 18.4% (95% CI: 8.8–28.0%) in Florida and 15.5% (95% CI: 11.0–20.1%) in New York. Rates of hospitalization for stroke were reduced by 18.1% (95% CI: 9.3–30.0%) in Florida. The few local comprehensive laws in Oregon were not associated with reductions in AMI or stroke statewide. *Conclusion*. Comprehensive smoke-free air laws are an effective policy tool for reducing the burden of AMI and stroke.

## 1. Introduction

Substantial evidence has accumulated showing that exposure to secondhand tobacco smoke is a serious and preventable public health hazard [1]. A complex mixture of particles and gases, secondhand smoke is associated with 38,000 deaths per year in the United States from coronary heart disease and lung cancer in nonsmokers [2]. Comprehensive smoke-free air laws successfully reduce secondhand smoke exposure among workers in smoke-free establishments [3] and among the general population [4]. A comprehensive review by the Institute of Medicine [5] concluded that there is a causal relationship between secondhand smoke exposure and cardiovascular disease, and smoke-free air laws that effectively reduce exposure to secondhand smoke reduce the likelihood of a cardiovascular event. Three meta-analyses [6–8] found an average reduction of 11% to 17% in cardiovascular hospitalizations or mortality following enactment of a smoke-free air law.

This study investigates the impact of smoke-free air laws in three US states—Florida, New York, and Oregon—on county-level rates of hospitalization for acute myocardial infarction (AMI) and stroke. We update a previously published estimate of reductions in AMI hospitalizations in New York [9] with additional data and provide the first estimates of the impact of smoke-free air laws in Florida and Oregon.

Florida, New York, and Oregon differ from each other in terms of their population demographics and experiences with smoke-free air laws, providing an opportunity to study the impact of similar laws in different settings. Florida enacted a statewide smoke-free air law in July 2003 banning smoking in all workplaces and restaurants, but exempting free-standing bars. Florida had no county-level comprehensive smoke-free air laws in place before the statewide law was enacted. New York also passed a statewide smoke-free air law in July 2003, covering free-standing bars in addition to workplaces and restaurants. New York's 2003 comprehensive law followed significant local-level smoke-free air policy enactment, such that by 2003, 75% of New York's population was covered by local laws stronger than an earlier state law passed in 1985. The comprehensive local laws in New York included a law banning smoking in all workplaces, restaurants, and bars in New York City that was enacted in March 2003. During the time period covered by this study, Oregon did not have a statewide smoke-free air law. A modest worksite law, enacted in 2001, excluded bars and bar areas within restaurants. Two Oregon localities enacted comprehensive laws during the study period. The cities of Corvallis and Eugeneenacted smoke-free air laws in 1998 and 2000, respectively. These ordinances were grandfathered in when the weaker statewide law passed, but preemption barred new comprehensive local laws from being passed after 2001. Oregon has subsequently enacted a statewide workplace, restaurant, and bar smoke-free air law that became effective in January 2009. 

## 2. Methods 

### 2.1. Data

We obtained data on hospital admissions for AMI and stroke from a comprehensive administrative database maintained by the department of health in each state. All nonfederal public and private hospitals certified for inpatient care are required to submit patient data, including diagnoses, to the central database. We derived admission rates from the diagnosis established at discharge. When AMI or stroke was a secondary diagnosis, it was not used in the calculation of the admission rate. Data from Florida were available on a quarterly basis, whereas data from New York and Oregon were available monthly. The number of years of available data from each state varied from 9 years for Oregon (January 1998–December 2006) to 12 years for New York (January 1995–December 2006) and 17 years for Florida (Quarter 1 1990–Quarter 4 2006). New York data from 1995 to 2004 are the same data used in Juster et al. [9]. An additional 24 months of data from New York are analyzed here.

The International Classification of Diseases, Ninth Revision, Clinical Modification (ICD-9-CM) diagnostic codes 410.00–410.99 identify admissions associated with AMI, and diagnostic codes 430.00–438.99 identify admissions associated with stroke. The number of hospital admissions associated with AMI and stroke for persons aged 35 or older for the years available was extracted for each county in each state, monthly in New York and Oregon and quarterly in Florida. We combined the number of hospital admissions with county population data to calculate the monthly (quarterly in Florida) rate of hospital admissions for each health condition. Rates were age-adjusted to the 2000 U.S. standard population. Age-adjusting the hospital admission rates controls for differing age distributions of the populations across the three states. 

Information about local smoking restrictions was purchased from the Americans for Nonsmokers' Rights Foundation Local Tobacco Control Ordinance Database (http://www.no-smoke.org/). The database describes all municipal smoking bans and includes dates of enactment and implementation and specific restrictions and prohibitions of each. Counties are classified into one of three mutually exclusive categories based on the strength of the smoking ban in effect in the county in a given month or quarter. A county or state was considered to have a comprehensive smoke-free air law if the law prohibits smoking in all worksites, including restaurants, bars, and other hospitality venues with few or no exceptions. Florida's 2003 statewide law was considered comprehensive for this analysis despite an exclusion for free-standing bars. Moderate laws were defined as those that restrict smoking in most worksites but provide little or no protection in restaurants and other hospitality venues. A county was considered to have no smoke-free air law if it did not have a moderate or a comprehensive law in place. Counties that had smoke-free air restrictions that applied only to municipal buildings were considered to have no smoke-free air law. Date of enactment for all laws was rounded to the nearest month or quarter.

### 2.2. Statistical Analyses

Multiple regression analysis was used to model the county-level age-adjusted hospital admission rates for AMI and stroke, separately. All analyses were conducted using Stata 11 [10]. The key explanatory variables in all models are an indicator for comprehensive smoke-free air law, an indicator for moderate smoke-free air law, and interactions of these terms with a linear time trend. The smoke-free air indicators are interpreted as the main effect of comprehensive and moderate smoke-free air laws on hospital admissions, measuring a one-time, immediate increase or decrease in rates at the time the law was enacted. The interaction between the smoke-free air law main effects and the time trend measures continued rate changes following the implementation of the law. Each model also includes an indicator for month (quarter for Florida) to control for seasonal effects. Unobserved, time-invariant county-level factors that are correlated with rates of cardiovascular disease risk and other conditions were controlled for by county indicator variables. To control for county-specific secular changes over time, we included interactions of the county indicator variables with the linear time trend. 

Estimated regression coefficients were used to predict the number of hospital admissions for AMI and stroke averted as a result of implementation of comprehensive smoke-free air laws. We first predicted monthly (or quarterly for Florida) rates of hospital admissions using the full model; this is the baseline case. We then set the comprehensive smoke-free air law indicator and comprehensive law-time interaction coefficients equal to zero beginning the month (or quarter) when a comprehensive smoke-free air law went into effect in each county and repredicted hospitalization rates; this is the counterfactual case. Because the regression coefficients for the comprehensive law main and interaction effects are generally negative, zeroing them out results in higher predicted rates of AMI and stroke hospitalizations compared to the base case. The difference between the base case and the counterfactual case is the amount that hospitalization rates were reduced by the implementation of comprehensive smoke-free air laws. We converted the rates into number of age-adjusted events by multiplying the estimated rate by the population of adults aged 35 or older in the given county and time period. We estimated 95% confidence intervals for the number of hospitalizations averted using the *bootstrap* command in Stata 11 [10]. 

## 3. Results 

Regression results for AMI hospitalization rates are reported in Table 1. The main effect for comprehensive smoke-free laws was statistically significant in New York (*b* = −1.483, *P* < 0.05) and marginally significant at the 10% level in Florida (*b* = −4.377, *P* < 0.10). The interaction between the comprehensive smoke-free air law and time is significant for Florida (*b* = −2.514, *P* < 0.01) and New York (*b* = −0.251, *P* < 0.01), suggesting that hospitalization rates for AMI decrease steadily over time after implementation of a comprehensive smoke-free air law. Moderate smoke-free laws were in effect in communities in New York and Oregon before comprehensive laws were enacted in those states. The moderate law main effect was not significant in New York, but it was significant and positive in Oregon (*b* = 3.846, *P* < 0.05). The interaction between moderate laws and time was negative and significant in New York (*b* = −0.124, *P* < 0.05) and Oregon (*b* = −0.242, *P* < 0.01). 

Results for stroke hospitalization rates are reported in Table 2. Florida's statewide comprehensive smoke-free air law is associated with a significant reduction in stroke hospitalization rates, both immediately at the time of implementation (main effect *b* = −16.194, *P* < 0.01) and over time (interaction effect *b* = −2.105, *P* < 0.01). In New York, local moderate smoke-free laws are associated with a significant increase in stroke hospitalization rates for both the main effect (*b* = 1.848, *P* < 0.01) and the interaction with the monthly time trend (*b* = 0.098, *P* < 0.01). In Oregon, moderate smoke-free laws are significantly associated with a decrease in stroke hospitalization rates over time (*b* = −0.122, *P* < 0.01). 

Results from the counterfactual analysis are presented in Table 3. In Florida, the statewide law restricting smoking in all workplaces and restaurants is associated with reductions in AMI hospitalizations of 18.4% (95% CI: 8.8–28.0%) and stroke hospitalizations by 18.1% (95% CI: 9.3–30.0%) over the time period from Quarter 3 2003 through Quarter 4 2006, a span of just over 3 years. On an age-adjusted basis, this is equivalent to approximately 32,425 (95% CI, 15,478–49,373) averted AMI cases and 44,485 (95% CI: 22,745–66,224) averted stroke cases. Annually, this is a decline of approximately 5.3% for AMI and 5.2% for stroke hospitalizations in Florida. 

New York's comprehensive statewide smoke-free air law lowered AMI hospitalizations by 15.5% (95% CI: 11.0–20.1%) between March 2003 and December 2006, an average annual reduction of 4.4%. This is equivalent to 28,649 (95% CI: 20,292–37,006) averted hospitalizations on an age-adjusted basis. Other effects were not associated with significant reductions in hospitalizations. 

## 4. Discussion

This paper updates a previous estimate of the impact of New York's comprehensive statewide smoke-free air law on AMI and stroke hospitalization rates [9] and examines the impact of similar laws in Florida and Oregon. More than 3 years after the comprehensive smoke-free laws went into effect, rates of hospitalization for AMI were significantly reduced by 18.4% in Florida and 15.5% in New York, and stroke hospitalization rates in Florida were reduced by 18.1%. Failure to detect a significant effect of comprehensive smoke-free laws in Oregon derives from the fact that only a few communities in Oregon had such laws during the period of this study. 

The 24 months of additional data from New York used in this study have strengthened the results for AMI hospitalizations reported previously for New York [9]. In the earlier paper, the point estimate for the main effect of comprehensive smoke-free laws was equal to −0.80 and not statistically significant. In this study, the main effect for comprehensive smoke-free laws is −1.483 and statistically significant. This suggests that the AMI hospitalization rate was immediately reduced after New York's comprehensive smoke-free laws went into effect, in addition to the gradual reduction over time suggested by the statistically significant interaction of comprehensive smoke-free laws with time, which is a similar magnitude (−0.32 versus −0.25) in both studies. 

Large and significant reductions were found in Florida for stroke hospitalizations, whereas none were detected in New York. One explanation is that the burden of stroke is greater in Florida than New York, providing a greater potential for improvement. Florida has a stroke prevalence rate of 2.7%, compared with 2.0% for New York, and a higher rate of mortality from cerebrovascular disease at 51.4 per 100,000 compared with 42.5 per 100,000 in New York [11]. It is also possible that population differences in age and race/ethnicity between New York and Florida contributed to these results. Nationally, the cerebrovascular disease mortality rate, which includes stroke, of non-Hispanics is approximately three times that of Hispanics (47.0 versus 14.6 deaths per 100,000, resp.), while Whites and African Americans have roughly equal cerebrovascular mortality rates (44.0 versus 38.7 deaths per 100,000, resp.) [12]. In 2010, 13.5% of New York's population was age 65 and older, 15.9% were African-American, and 17.6% were Hispanic (http://quickfacts.census.gov/qfd/states/36000.html). In Florida, 17.3% were age 65 and older, 16.0% were African-American, and 22.5% were Hispanic (http://quickfacts.census.gov/qfd/states/12000.html). Use of age-adjusted hospitalization rates will have ameliorated the effect of age differences across states, and the difference in race/ethnicity is not that great. Still, the difference in results for stroke between the two states is striking and remains unexplained. Perhaps the results in New York are capturing an increase in some causal factor for stroke itself or an increase in its diagnosis that is not occurring simultaneously in Florida. Similar studies in the future should consider incorporating more controls to explicitly account for differences in rates of disease by race/ethnicity. 

We have attempted to be conservative in interpreting our findings. As with any study relying solely on observational data, there are limitations to the strength of association that can be inferred. Hospitalization rates for AMI and stroke have been declining for many years. While some of this decline is likely due to adoption of smoke-free air laws, some of it may be attributable to changes in other factors affecting cerebrovascular and cardiovascular disease more generally, such as declining prevalence of smoking and reductions in daily cigarette consumption among remaining smokers, increased public health focus on obesity and physical activity, improvements in air quality, and other factors. The secular time trends and area fixed effects in our models control for many of these effects, but imperfectly. Because the data used are aggregated, county-level rates, we are unable to assess exposure to secondhand smoke among at-risk individuals or to differentiate between current smokers and nonsmokers. To the best of our knowledge, county-level data on secondhand smoke exposure or smoking rates among adults over time do not exist in any of the states considered in this paper. 

## 5. Conclusions

We provide evidence for significant reductions in hospital admissions for stroke and AMI in Florida and significant reductions in admissions for AMI in New York following implementation of comprehensive smoke-free air laws. These results are consistent with a growing body of literature suggesting a direct association between laws banning smoking in public places and improvements in public health. A great deal of progress has been made in the past 10 years in the adoption of comprehensive smoke-free air laws in the United States, but still only 48% of the U.S. population is currently covered [13]. Given the rapidly rising cost of health care and wide public support for smoke-free air laws, comprehensive smoking bans should be considered by all state and local governments as an effective measure to improve health and reduce health care costs. 

## Figures and Tables

**Table 1:** Single-state regression models, age-adjusted rate of hospital admissions for acute myocardial infarction (AMI).

Independent variable	Florida	New York	Oregon
Comprehensive smoke-free air law	−4.377^∗^	−1.483^∗∗^	4.306
	(2.243)	(0.675)	(5.400)
Comprehensive smoke-free air law × time interaction	−2.514^∗∗∗^	−0.251^∗∗∗^	−0.126
	(0.225)	(0.022)	(0.173)
Moderate smoke-free air law	—	−0.882	3.846^∗∗^
		(0.569)	(1.771)
Moderate smoke-free air law × time interaction	—	−0.124^∗∗^	−0.242^∗∗∗^
		(0.040)	(0.056)
Time (monthly linear trend)	—	−0.061^∗∗∗^	−0.046
		(0.013)	(0.059)
Time (quarterly linear trend)	−0.227^∗∗^	—	—
	(0.094)		
Constant	128.335^∗∗∗^	39.288^∗∗∗^	30.633^∗∗∗^
	(4.124)	(1.131)	(4.478)
Number of observations	4,556	8,928	3,888
Adjusted *R* ^²^	0.469	0.353	0.138

^∗^ *P* < 0.10, ^∗∗^ *P* < 0.05, ^∗∗∗^ *P* < 0.01.

Standard error in parentheses.

Data for Florida are quarterly from 1990 to 2006, data for New York are monthly from 1995 to 2006, and data for Oregon are monthly from 1998 to 2006. All models include county indicators and county × time interactions.

**Table 2:** Single-state regression models, age-adjusted rate of hospital admissions for stroke.

Independent variable	Florida	New York	Oregon
Comprehensive smoke-free air law	−16.194^∗∗∗^	−0.724	−1.776
	(2.380)	(0.551)	(3.183)
Comprehensive smoke-free air law × time interaction	−2.105^∗∗∗^	0.025	−0.157
	(0.269)	(0.018)	(0.131)
Moderate smoke-free air law	—	1.848^∗∗∗^	−0.578
		(0.483)	(1.321)
Moderate smoke-free air law × time interaction	—	0.098^∗∗∗^	−0.122^∗∗∗^
		(0.034)	(0.046)
Time (monthly linear trend)	—	−0.067^∗∗∗^	−0.170^∗∗∗^
		(0.013)	(0.064)
Time (quarterly linear trend)	−0.119	—	—
	(0.138)		
Constant	176.250^∗∗∗^	49.722^∗∗∗^	50.128^∗∗∗^
	(6.155)	(1.100)	(5.413)
Number of observations	4,556	8,928	3,888
Adjusted *R* ^²^	0.475	0.329	0.134

^∗^ *P* < 0.10, ^∗∗^ *P* < 0.05, ^∗∗∗^ *P* < 0.01.

Standard error in parentheses.

Data for Florida are quarterly from 1990 to 2006, data for New York are monthly from 1995 to 2006, and data for Oregon are monthly from 1998 to 2006. All models include county indicators and county × time interactions.

**Table 3:** Estimated total reductions in hospital admissions for acute myocardial infarction (AMI) and stroke attributed to implementation of comprehensive smoke-free air laws.

Diagnosis	Percentage		
	(95% CI)		
	Number of age-adjusted cases		
	(95% CI)		
	Florida^a^	New York^b^	Oregon
AMI	18.4%	15.5%	NS
	(8.8%–28.0%)	(11.0%–20.1%)	
	32,425	28,649	
	(15,478–49,373)	(20,292–37,006)	
Stroke	18.1%	NS	NS
	(9.3%, 30.0%)		
	44,485		
	(22,745–66,224)		

NS: not significant.

^a^Between Quarter 3 2003 and Quarter 4 2006.

^b^Between March 2003 and December 2006.
